# Advances in the Use of Immunotherapy in Oncology

**DOI:** 10.6004/jadpro.2017.8.3.2

**Published:** 2017-04-01

**Authors:** Anthony J. Olszanski, Laura J. Zitella

**Affiliations:** 1 Fox Chase Cancer Center, Philadelphia, Pennsylvania;; 2 Stanford Cancer Institute, Palo Alto, California

## Abstract

Innovations in cancer immunotherapy have created new options for patients and avenues for clinicians to practice precision medicine.

One of the major breakthroughs in cancer therapy in the past 50 years, immunotherapy represents a new beginning for medical oncology, according to Anthony J. Olszanski, MD, RPh, of the Fox Chase Cancer Center, Philadelphia.

"Immunotherapy is changing the way that oncologists think about patients and the way that patients behave in our clinics," said Dr. Olszanski. "It’s producing wonderful and durable responses in a variety of different diseases, and patients are thriving years after treatment."

At the 2016 JADPRO Live conference, Dr. Olszanski was joined by Laura J. Zitella, MS, RN, ACNP-BC, AOCN®, of the Stanford Cancer Institute, Palo Alto, California, to discuss treatment management for patients receiving immunotherapeutic agents and the role of testing for programmed cell death protein ligand (PD-L1) in advance of starting treatment. Dr. Olszanski and Ms. Zitella also touched on the concept of pseudoprogression and its possible impact on treatment.

Although there are many different types of immunotherapy, including cytokines, oncolytic viruses, tumor-infiltrating lymphocytes, vaccines, and chimeric antigen receptor (CAR) T-cell therapy, the speakers focused on immune checkpoint inhibitors, which are the most commonly used clinical immunotherapeutics today.

## IMMUNE CHECKPOINT INHIBITORS

The extraordinarily complicated immune system consists of highly specialized cells designed to detect and destroy pathogens, explained Ms. Zitella. With their ability to recognize and attack cells that are old, diseased, or cancerous, T cells play a critical role in protecting the body against cancer. After the immune system has been activated to fight infection, so-called immune checkpoints, such as cytotoxic T-lymphocyte–associated protein 4 (CTLA-4) and programmed cell death protein 1 (PD-1), are then used to "turn it off" (Luke & Ott, 2015).

"You don’t want to keep the immune system ’turned on’ forever," said Ms. Zitella. "CTLA-4 and PD-1 are part of the natural feedback loop to suppress the immune response. They are the brakes."

Although immune checkpoints exist as a normal function to protect self from inflammation, autoimmunity, allergy, hypersensitivity, pregnancy, and allograph, they can also allow tumors to evade the immune system by inducing immune tolerance. Recently, however, researchers have begun to exploit this process in the treatment of cancer. Moreover, said Ms. Zitella, because immune checkpoint inhibitors operate at different stages of the immune response, there are several opportunities for therapeutic agents.

As Dr. Olszanski indicated, there has been a huge upswing in the number of US Food and Drug Administration (FDA) approvals in the past 5 years. Ipilimumab (Yervoy), an anti–CTLA-4 agent, was the first monoclonal antibody approved for advanced melanoma and is now also indicated for adjuvant melanoma. Nivolumab (Opdivo) and pembrolizumab (Keytruda), anti–PD-1 antibodies, have been approved for a number of diseases (e.g., advanced melanoma, head and neck cancer, and lung cancer in the second line; nivolumab is also indicated for advanced renal cell carcinoma and classical Hodgkin lymphoma). The newest approved agent, atezolizumab (Tecentriq), an anti–PD-L1 antibody, has been approved for second-line lung cancer and bladder cancer. A recent approval, said Dr. Olszanski, is for pembrolizumab in the first-line setting of non–small cell lung cancer (NSCLC) if PD-L1 expression is over 50%.

"Immunotherapy has been effective in a wide range of tumor types (Figure 1), and we’re using a lot of immunotherapy on clinical trials these days," said Dr. Olszanski. "In NSCLC, which is so hard to treat, we now have a 15%–20% response rate, but what’s really amazing is that in some of the combinations of nivolumab plus ipilimumab, we’re seeing durable responses in NSCLC" (Champiat et al., 2016).

**Figure:**
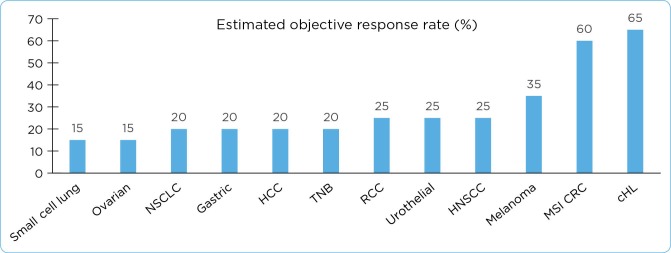
Immunotherapy is effective in a wide range of tumor types. NSCLC = non–small cell lung cancer; HCC = hepatocellular carcinoma; TNB = triple-negative breast cancer; RCC = renal cell carcinoma; HNSCC = head and neck squamous cell carcinoma; MSI CRC = microsatellite instability in colorectal cancer; cHL = classical Hodgkin lymphoma. Adapted from Champiat et al. (2016); Chiou and Burotto (2015).

"Furthermore, in colorectal cancer with high microsatellite instability (MSI), which accounts for 15% of that population, there’s a 60% overall response rate," he added. "That’s really exciting. You have to find these patients who are MSI-high." Patients with high microsatellite instability are prone to tumors with a large number of somatic mutations, which may predict the efficacy of PD-1 inhibition.

## IS PD-L1 TESTING NECESSARY?

Testing of PD-L1 remains a controversial topic. Although current data suggest that higher expression of PD-L1 predicts a greater likelihood of response to PD-1 or PD-L1 inhibition, patients with a low expression of PD-L1 may also respond to treatment. Testing of PD-L1 is currently indicated for pembrolizumab in NSCLC, but there is continuing research in that area, Ms. Zitella said.

"It’s hard to know what to do in those situations," she commented. "We don’t want to deny a patient a potentially effective therapy based on his or her PD-L1 testing."

Dr. Olszanski remarked upon the many challenges associated with the PD-L1 biomarker. Immunohistochemistry assays differ among testing platforms and might actually yield different results. In addition, he said, testing confirms positivity on tumor cells, but tumor-infiltrating lymphocytes could also stain positive. A standard testing threshold has not been established, and heterogeneity in biopsies can lead to discordant PD-L1 results within the same tumor. Finally, he added, PD-L1 expression may change due to pressure from prior therapies.

## MANAGEMENT OF SIDE EFFECTS

Side effects from immunotherapy differ dramatically from those that advanced practitioners are used to seeing with other agents, Ms. Zitella observed. "Jump-starting the immune system can induce an autoimmunity or reactivity against healthy cells, and this can cause side effects," she explained. "While we’re excited about these inhibitors, we are still learning best practices for early diagnosis and management of side effects."

According to Ms. Zitella, rash, fatigue, pruritus, and diarrhea are the most common side effects from PD-1/PD-L1 and CTLA-4 inhibitors, but immune-related adverse reactions can affect any tissue (Table 1; Champiat et al., 2016). Infrequently, she said, you can also see liver abnormalities, endocrinopathies, and pneumonitis. More rarely, she added, problems in other organs—encephalitis, pancreatitis, or nephritis, for example—may occur.

**Table 1:**
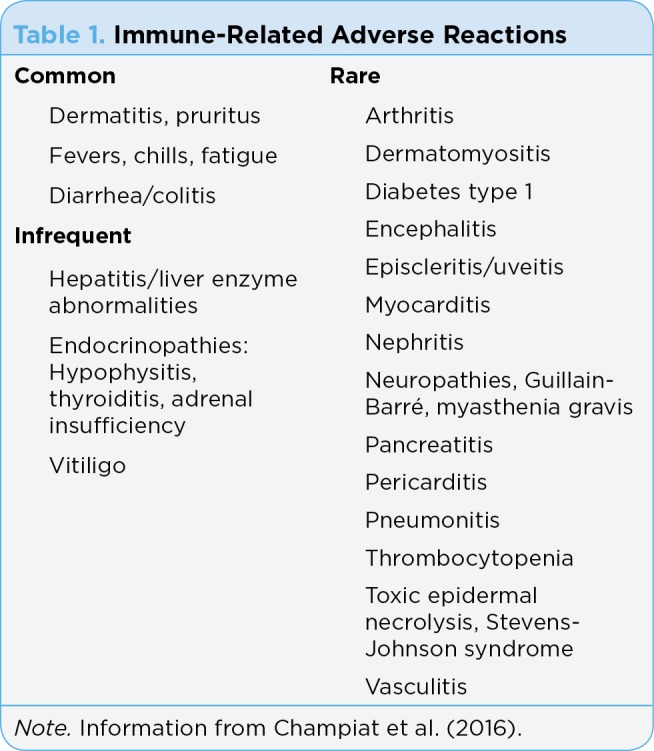
Immune-Related Adverse Reactions

"As much as we talk about the side effects," she said, "I want to emphasize that most of them are mild. These drugs are actually very well tolerated, especially when compared with the side effects of chemotherapy."

## TIMING OF TREATMENT-RELATED SIDE EFFECTS

Dr. Olszanski noted that the average time to onset of immune-related adverse events (irAEs)—should they occur—is approximately 6 to 12 weeks after initiation of therapy (Weber, Kähler, & Hauschild, 2012; Weber, Yang, Atkins, & Disis, 2015). They tend to emerge in the following order:

- Skin: after 2 to 3 weeks
- Gastrointestinal: after 5 to 6 weeks
- Hepatic: after 6 to 7 weeks
- Endocrine: after 8 to 9 weeks
- irAEs are rare after 24 weeks

"When patients start therapy," he said, "they expect side effects immediately, so it’s important to educate patients on what to report, especially if something happens 2 or 3 months into therapy."

Consultants and emergency room physicians require additional education as well, he added, because the side-effect profile is so different from that of chemotherapy. "It’s important for patients to have a wallet card to alert health-care providers that they’re on these drugs, especially those with metastatic lung cancer, because they are at risk for a lot of infections," Ms. Zitella added. "For example, if a patient treated with immunotherapy presents with shortness of breath, immune-mediated pneumonitis should be included in the differential diagnosis by emergency room physicians."

Symptoms of immune-mediated pneumonitis include shortness of breath, dry cough, and new or increasing oxygen requirements, but pneumonitis may also be detected on imaging alone. "You can always hold the immunotherapy and start steroids to be on the safe side," said Ms. Zitella. "If patients are having true immune-mediated pneumonitis, we typically see a response to steroids within the first week."

A management approach to irAEs is shown in Table 2. In general, said Ms. Zitella, the following signs require prompt evaluation: diarrhea, blood in the stool, fatigue, weight loss, nausea or vomiting, new rash, shortness of breath or cough, and any neurologic change. In some patients who have been on immunotherapy for more than 2 years, clinicians are also seeing arthralgia and neuropathy develop as very delayed side effects.

**Table 2:**
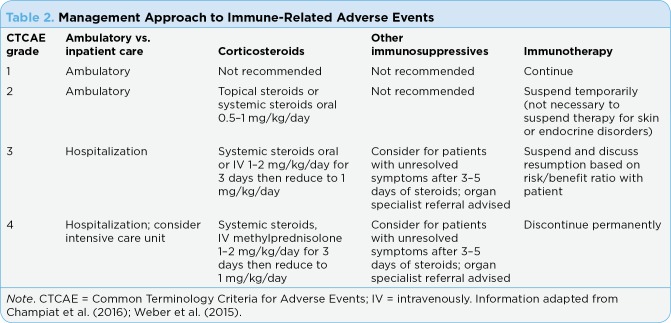
Management Approach to Immune-Related Adverse Events

"Generally speaking," said Ms. Zitella, "if irAEs are mild, you should treat with supportive care. If the adverse event is grade 2 or higher, you should use steroids. If it is grade 3 or 4, which requires hospitalization, you should use higher-dose steroids or intravenous (IV) steroids" (Champiat et al., 2016; Weber et al., 2015).

"It’s important to recognize that when we’re giving high-dose steroids, we’re suppressing the patient’s immune system," said Dr. Olszanski. "We have to remember that other opportunistic infections can occur."

"Many clinicians that treat solid tumors aren’t used to using high-dose steroids," Ms. Zitella added. "Patients need gastrointestinal prophylaxis against stress ulcers, assessment for oral thrush, and prophylaxis for *Pneumocystis jiroveci* pneumonia. In addition, blood glucose and blood pressure should be closely monitored."

Although there’s been obvious concern that the use of steroids may diminish the effectiveness of immunotherapy, according to Ms. Zitella, retrospective analysis of melanoma patients who were treated with steroids has shown no difference in the time to treatment failure or overall survival (Horvat et al., 2015).

## PSEUDOPROGRESSION

Pseudoprogression, which is characterized by an apparent increase in tumor burden (usually of at least 25% at imaging assessment), can be a real challenge for clinicians (Hodi et al., 2016). Pseudoprogression can be early (by week 12) or delayed (after week 12), but it is not very common, Ms. Zitella indicated.

"An increase in tumor size could be tumor infiltration with immune cells or inflammation," according to Ms. Zitella. "It can be very difficult to determine if this is progressive disease or pseudoprogression."

The traditional criteria for evaluating response are the Response Evaluation Criteria in Solid Tumors (RECIST), which take a maximum of five target lesions and assess their size; the appearance of any new lesion counts as progressive disease (Eisenhauer et al., 2009). Because of the aforementioned atypical responses with immunotherapy, however, researchers have devised immune-related response criteria (irRC), which consider a greater number of lesions, measure overall tumor burden, and allow for the addition of a few new lesions. With irRC, progressive disease is assessed based on the overall tumor burden rather than just a small number of target lesions, but, said Ms. Zitella, it is still an imperfect means of assessing response. In clinical practice, when faced with a patient who could have progressive disease and metastatic cancer with limited treatment options, she gives patients "the benefit of the doubt" that the change could reflect pseudoprogression.

"That’s what we’re seeing in clinical practice," she said. "If a patient is tolerating immunotherapy well, it’s generally continued."

Also important to remember, Ms. Zitella added, is that it takes time to generate a T-cell response. "We have to be careful about imaging patients too early," she cautioned. "Try to wait 8–12 weeks to allow the treatment to work before reassessing for response."

In summary, Ms. Zitella offered these "pillars" of immunotherapy management: understand the toxicity spectrum; educate patients; intervene early to prevent severe adverse effects; and monitor patients.

## UNANSWERED QUESTIONS AND FUTURE POSSIBILITIES

Due to the fact that these agents are still new, many unanswered questions remain. In addition to evaluating response, researchers are trying to determine whether biomarkers can predict response and how treatment should be optimally sequenced and combined—and that is just for those immune checkpoint inhibitors already on the market. Over the course of the next 5 to 10 years, commented Dr. Olszanski, a "huge volume" of new PD-1 and PD-L1 inhibitors will be approved, along with other immunotherapies, he predicted.

"Right now at my center, I’m investigating no fewer than 10 different checkpoint molecules," said Dr. Olszanski. "We’re only at the cusp of the beginning of immunotherapy, and really exciting times are ahead."
